# AS1411 Aptamer-Anionic Linear Globular Dendrimer G2-Iohexol Selective Nano-Theranostics

**DOI:** 10.1038/s41598-017-12150-8

**Published:** 2017-09-19

**Authors:** Pardis Mohammadzadeh, Reza Ahangari Cohan, Seyedeh Masoumeh Ghoreishi, Ahmad Bitarafan-Rajabi, Mehdi Shafiee Ardestani

**Affiliations:** 1 Department of Genetics and Molecular Biology, Isfahan University of Medical Sciences, Isfahan, Iran; 2 Department of Pilot Nanobiotechnology, New Technologies Research Group, Pasteur Institute of Iran, Tehran, Iran; 3 Department of Radiopharmacy, Faculty of Pharmacy, Tehran University of Medical Sciences, Tehran, Iran; 4 Echocardiography Research Center, Cardiovascular Interventional Research Center, Department Of Nuclear Medicine, Rajaie Cardiovascular Medical And Research Center, Iran University Of Medical Sciences, Tehran, Iran; 5 Department of Radiopharmacy, Faculty of Pharmacy, Tehran University of Medical Sciences, Tehran, Iran

## Abstract

Molecular theranostics is of the utmost interest for diagnosis as well as treatment of different malignancies. In the present study, anionic linear globular dendrimer G2 is employed as a suitable carrier for delivery and AS1411 aptamer is exploited as the targeting agent to carry Iohexol specifically to the human breast cancer cells (MCF-7). Dendrimer G2 was prepared and conjugation of dendrimer and aptamer was carried out thereafter. Based on the data yielded by AFM, morphology of smooth and spherical non-targeted dendrimer changed to the rough aspherical shape when it conjugated. Then, conjugation was confirmed using DLS, ELS and SLS methods. Toxicity on nucleolin positive MCF-7 cells and nucleolin negative HEK-293 cells was assessed by XTT and apoptosis/necrosis assays. *In vitro* uptake was determined using DAPI-FITC staining and ICP-MS methods. *In vivo* studies including *in vivo* CT imaging, pathology and blood tests were done to confirm the imaging ability, bio-safety and targeted nature of the Nano-Theranostics *in vivo*. In a nutshell, the prepared construction showed promising effects upon decreasing the toxicity of Iohexol on normal cells and accumulation of it in the cancer tumors as well as reducing the number of cancer cells.

## Introduction

Breast cancer is one of the most frequently diagnosed and leading cause of cancer-related deaths among women worldwide^1^. Cancer can be hard to detect but early diagnosis increases the chance of effective treatment in some types of cancer. Although biopsies (such as tissue biopsy, needle biopsy, liquid biopsy, etc.) has remained as a gold-standard for diagnosis of many malignancies so far, it is expensive, time-consuming and utilizing invasive methods that cause health risks^2–5^. Conventional imaging procedures make use of non-targeted imaging agents (X-ray attenuation by barium or iodinated based agents, MR signal enhancement by superparamagnetic or paramagnetic agents, such as gadolinium-based contrast agents or ultrasound scattering and frequency shift by microbubble contrast agents) which passively flow through the internal body conduits and delineate anatomical and physiochemical changes in tissues. However, unfortunately, these agents have various undesirable effects, such as mild late allergic reactions, anaphylactic reactions (acute allergic reactions) which are life threatening emergencies or contrast-induced nephropathy (CIN) which is a famous adverse reaction of intravenously or intra-arterially delivered contrast materials^6–9^. Currently, numerous efforts have been done to reduce side effects and increase the precision of the procedure by means of nanoparticles and targeting agents. Recent advances in molecular imaging have proposed the utilization of alternative non-invasive, more precise and real-time imaging methods, using specific molecular probes (aptamers^10^, antibodies^11^, peptides, small molecules^12^, etc.) for studying the pathological, biochemical and physiological changes of an event at cellular and molecular level in a living organism^13–15^. These imaging techniques have become a key tool, not only to early detection of many cancers but to determine the stage, precise location, size and shapes of tumors and occurrence of metastasis, in order to help treat cancer, evaluate the effects of cancer therapies or examine recurrence of the disease^16,17^.

There are numerous types of nanoparticles have been used in various studies up to now (gold nanoparticles, silver nanoparticles, magnetic nanoparticles, dendrimers, etc.). These chemically synthesized compounds can trigger controlled release of materials (drugs, contrast agents, genes, etc.) they carry off, specific bio-distribution and targeting and abolish drug resistance^18^. The benefit of using nanoparticulate contrast media carriers compared to the commonly used organic imaging molecules is the ability to provide high-resolution images, long-term and multimodal imaging (SPECT/CT, MRI/optical, PET/CT, etc.) or for theranostics purposes^16^. Dendrimers are 3D nanostructures with many potential applications, including drug delivery and medical imaging modalities. Dendrimers are multi-branched nanoparticles with different categories. Some are positive charged for instance, PAMAM and some are negative charged such as Anionic globular dendrimers. The current study has utilized Anionic Linear Globular Dendrimer Generation 2 (ALGDG2) as a nanocarrier. ALGDG2 was introduced for the first time by Namazi and Adeli (2005)^19^. Easy synthesis, low molecular weight, well-known functional groups on the surface of the spherical particle, monodispersity, high purity and hydrophilicity, increased permeability to cancerous cells and most importantly low toxicity with low immune system stimulation when injected locally^20^ for its biocompatible PEGylated core and biodegradable sidelong citric acid groups made ALGDG2 a suitable carrier for a range of drugs and agents, such as anticancer^21^, antiviral^22^, antibacterial drugs^23^ and imaging agents^24^.

Application of active targeted conjugates besides other techniques can result in the more efficient imaging. Antibodies, peptides and aptamers are the most known biological ligands of cell surface biomarkers which can be used to target specific cells^25^. However, aptamers have some benefits over their protein counterparts. They have uniform activity regardless of batch synthesis, versatile selection process, wide variety of chemical modifications to modulate diverse functions, lack of immunogenicity, ease of chemical synthesis, unlimited shelf-life and high binding specificity and affinity in the low nanomolar/high picomolar range^26^. Aptamer decorated nanoparticles, which can be bound to target cell-surface biomarkers through shape complementarity by way of non-covalent bonds, provide a sensitive and specific targeted drug delivery, imaging and diagnostic modalities for both *in vitro* and *in vivo* applications^27^. In this study, AS1411 aptamer has been exploited which was previously discovered by Bates, *et al*. (2009)^28^, as a targeting agent. AS1411 aptamer is a 26 bp single stranded DNA oligonucleotide with high affinity and specificity to a putative surface biomarker, nucleolin, which is an overexpressed protein on numerous cancer cells regardless of tissue origin^29,30^, such as breast, colon, lung, prostate, gastric, etc. Nucleolin major role is in controling of cell proliferation, apoptosis and promoting cell-survival. The short length, G-rich four stranded (quadruplex) structure of AS1411 made it more stable against the serum nucleases and pH fluctuations and increased the cellular uptake efficacy^28^. Behrouz, *et al*. previously made use of AS1411 aptamer and PAMAM dendrimer conjugates as a targeted nanocarrier of 5-fluorouracil for the treatment of gastric cancer^31^. On the other hand, similar to other positively charged nanoparticles, PAMAM is toxic to normal cells due to its disruptive interaction with normal cells’ plasma membrane^32^.

Iohexol (trade name: omnipaque^TM^ or exypaque) is a commonly used nonionic water-soluble iodinated contrast agent for CT scan. Like other conventional contrast agents, Iohexol can cause some adverse effects such as CIN on patients^33^. Furthermore, due to the small size of iodine molecule, iodinated contrast agents are always being cleared by the kidneys so fast and thus, imaging is possible only in a short period of time^34^. In many other researches, scientists focused on utilizing different kinds of gold nanoparticles to achieve high resolution CT images and provide photothermal therapy. Although gold nanoparticles have shown promising outcomes in diagnosis and treatment of many malignancies in recent studies, they encounter some important challenges, such as price and availability because Au (gold) is a mineral nonrenewable resource that cannot be replenished. Therefore, It is obvious that while designing a new drug, it must be considered that every patient in the society with different socioeconomic status should have access to and the power of purchasing the drug. Moreover, There is no consensus on the toxicity of gold nanoparticles. However, a lot of researches are in agreement with the toxic effects of gold nanoparticles on human body. On the other hand, even if they are nontoxic, these kinds of nanoparticles are not fully bio-compatible (bio-inert)^35,36^.

**Figure 1:**
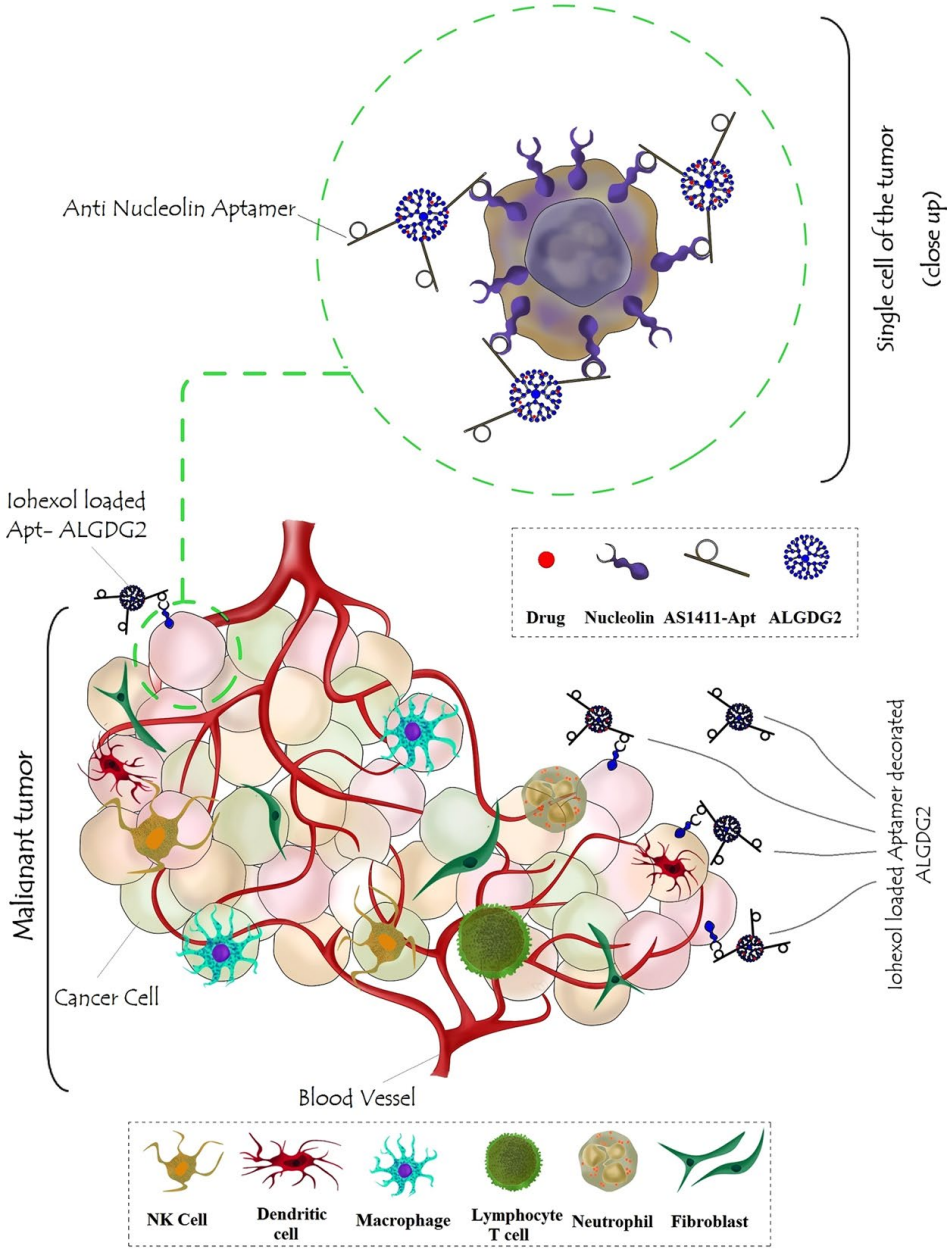
Graphical scheme which demonstrated total novelty and aim of the experiment.

In this research for the first time, controlled release, Iohexol cytotoxicity reduction on normal cells and targeting property to the cancer cells were employed by ALGDG2 which is targeted by AS1411 aptamer to cancer cells (graphical scheme: Fig. 1). This study focused on diminishing the cytotoxicity of the conventional contrast media (Iohexol) on normal cells and reducing the dosage of the drug which is needed for the imaging process, using a low molecular weight, non-cytotoxic and negative charged nanoparticle (ALGDG2).

## Results

### ALGDG2 and Apt-ALGDG2 conjugate synthesis confirmation and characterizations

**Figure 2:**
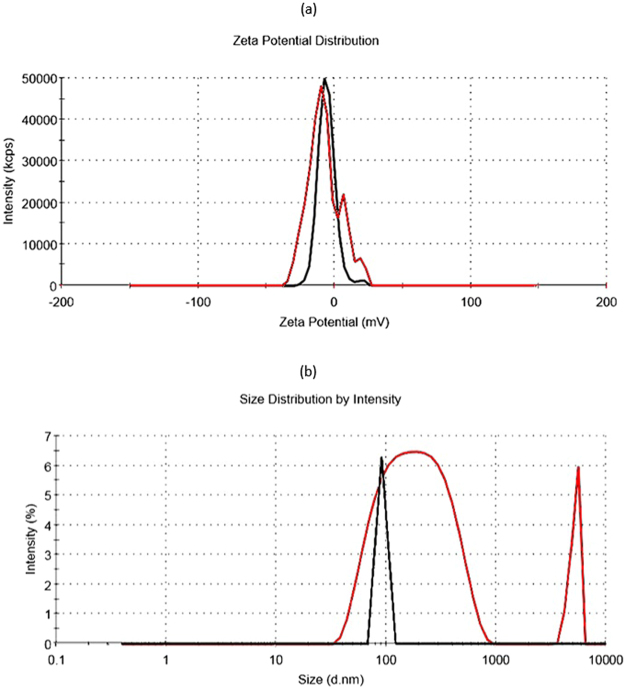
(**a**) Zeta potential of Apt-ALGDG2 conjugate (red) compared to ALGDG2 (black), (**b**) Hydrodynamic size distribution of Apt-ALGDG2 conjugate (red) and ALGDG2 (black).

**Figure 3:**
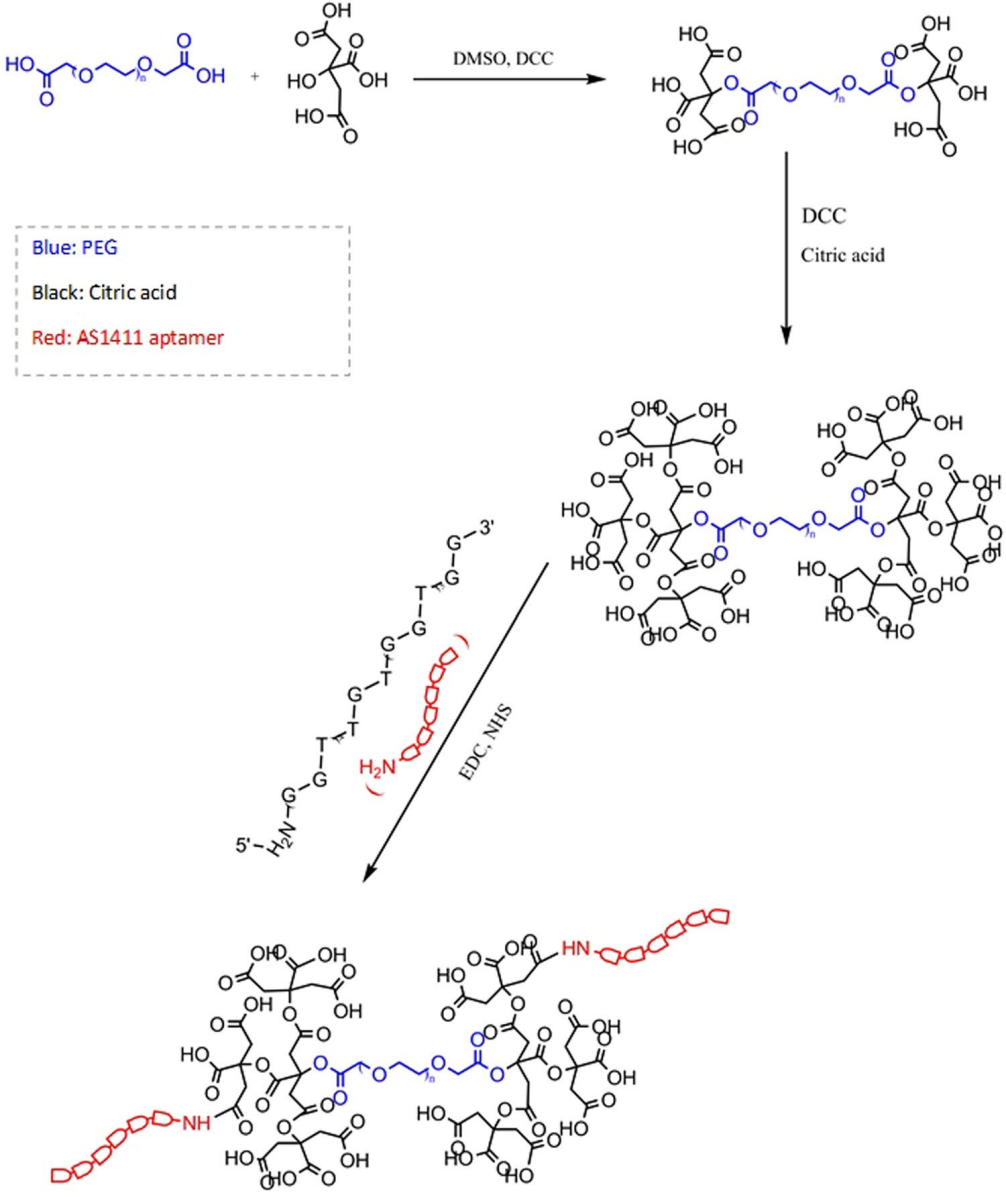
Schematic illustration of synthesis of ALGDG2 and Apt-ALGDG2 conjugate. Red: AS1411 aptamer, Blue: PEG and Black: citric acid.

The hydrodynamic size distribution (215.6 nm) and zeta potential (−11.6 mV) of Apt-ALGDG2 conjugate and hydrodynamic size distribution (91.3 nm) and zeta potential (−5.88 mV) of ALGDG2 were calculated and reported using dynamic light scattering (DLS) and electrophoretic light scattering (ELS). Comparative graphs are shown in (Fig. 2), respectively. ^1^HNMR, FT-IR and LC-MS graphs are presented in (Figure S1: S1a,b and c). The schematic illustration of the chemical synthesis process is shown in (Fig. 3).

### ALGDG2 and Apt-ALGDG2 conjugate morphological study

**Figure 4:**
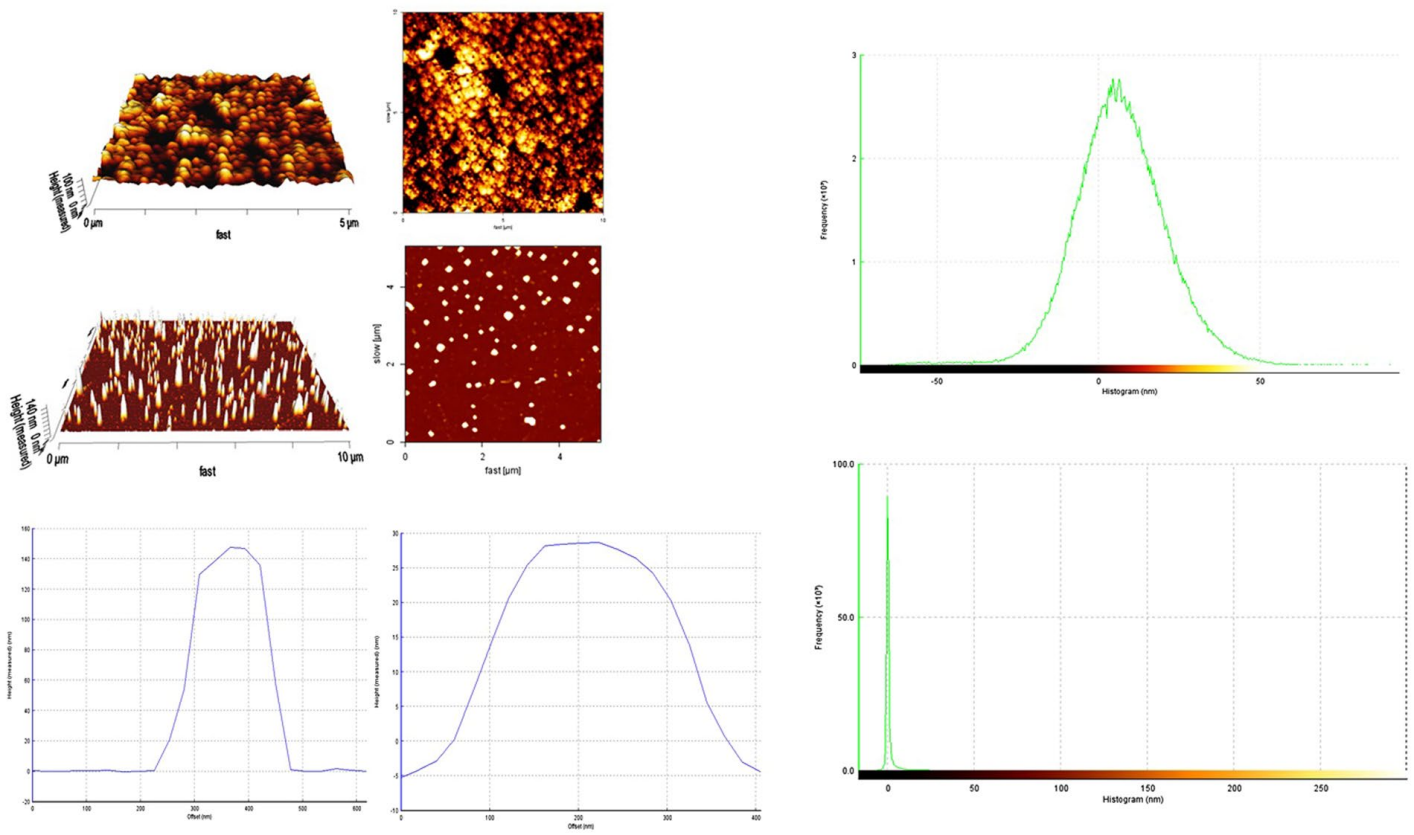
3D (left) and 2D (right) AFM images of ALGDG2 with average roughness Ra of 12.38 nm and RMS roughness Rq of 15.92.nm (up) and Apt-ALGDG2 conjugate with average roughness Ra of 59.38 nm and RMS roughness Rq of 62.52.nm (middle). Blue Plots compare the height (Y axis) to offset (X axis) measurements (nm) of conjugate (left) and ALGDG2 (right). The Green Histograms show the frequency (up = ALGDG2, Down = conjugate). Peak-to-valley roughness Rt of the ALGDG2 is 147.3 nm and the physical size is 0.31 × 0.26 µm. The related data for the conjugate are 316 nm and 0.33 × 0.39 µm respectively.

Using Atomic Force microscopy (AFM) by intermittent contact (Air) mode, the morphological status of the unconjugated ALGDG2 and Apt conjugated ALGDG2 were characterized and the 2D and 3D images of ALGDG2 spherical and smooth shape and Apt-ALGDG2 aspherical and rough shape are presented in (Fig. 4). The Graphs clearly indicate a dramatic increase in the height and offset of the conjugate compared to the dendrimer alone.

### Molar mass measurement

**Figure 5:**
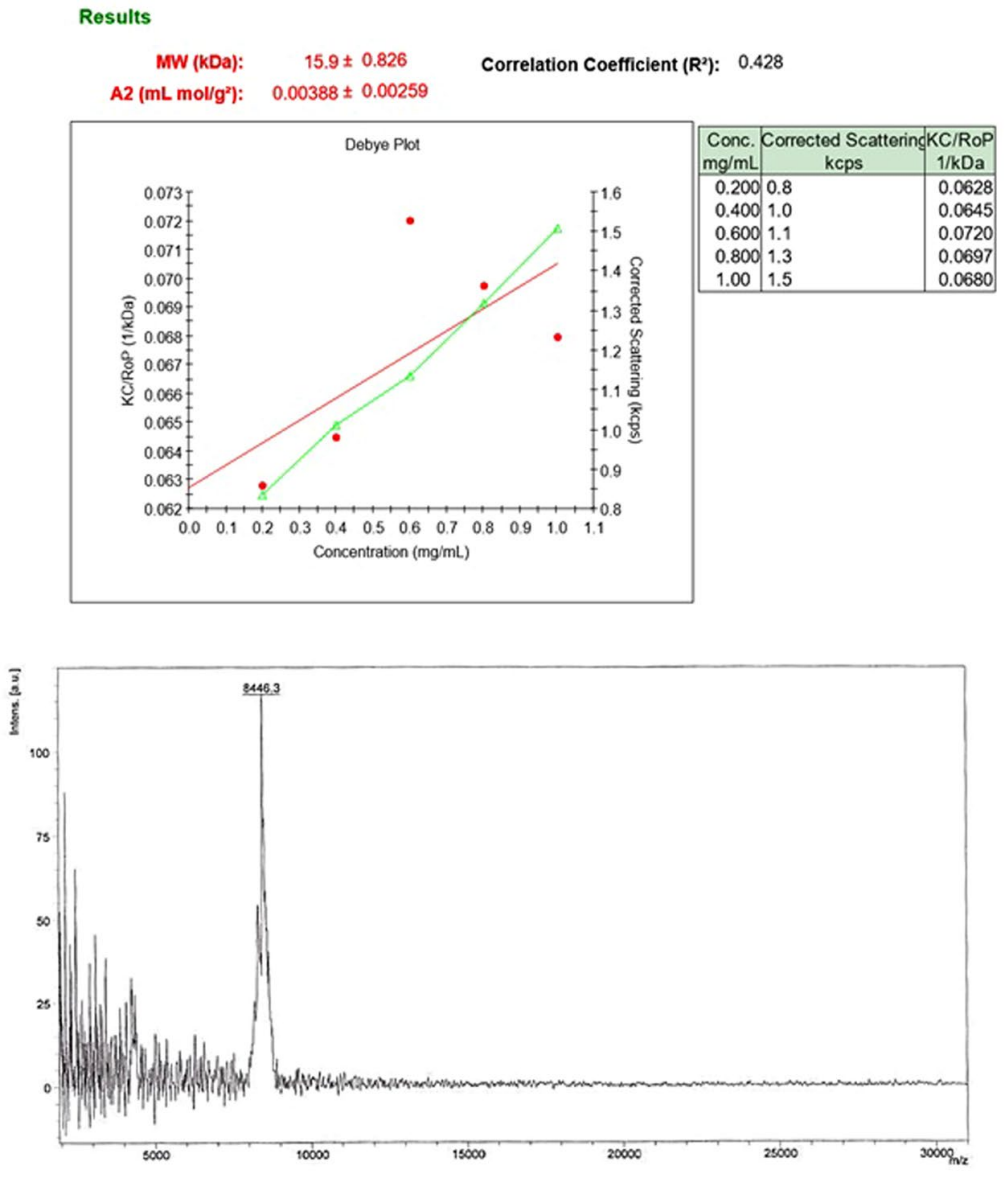
Debye plot of desired molar mass of Apt-ALGDG2 conjugate (up), MALDI-TOF mass Spectrometry of 5′ modified AS1411 aptamer (down).

The average molecular weight for the Apt-ALGDG2 conjugate was 15.9 ± 0.826 kDa. Molar mass of 5′-NH_2_ modified AS1411 aptamer was determined 8.444 kDa using MALDI-TOF mass spectrometry and both the related plots and all the data of SLS method including Debye plot are presented in (Fig. 5). Moreover, due to the previous studies^20^, the average molecular weight of intact ALGDG2 was reported 2 kDa. Comparing the molecular weights of ALGDG2, AS1411 aptamer and the conjugate, differences confirm the conjugation and the average efficacy of conjugation process is estimated to be 2:1 or 1:1 (Apt: ALGDG2) molar ratio per particle.

### Entrapment efficacy of ALGDG2

The amount of Iohexol encapsulated in ALGDG2 was found to be 34.6% by spectrophotometry.

### Apt-ALGDG2 conjugate stability

**Figure 6:**
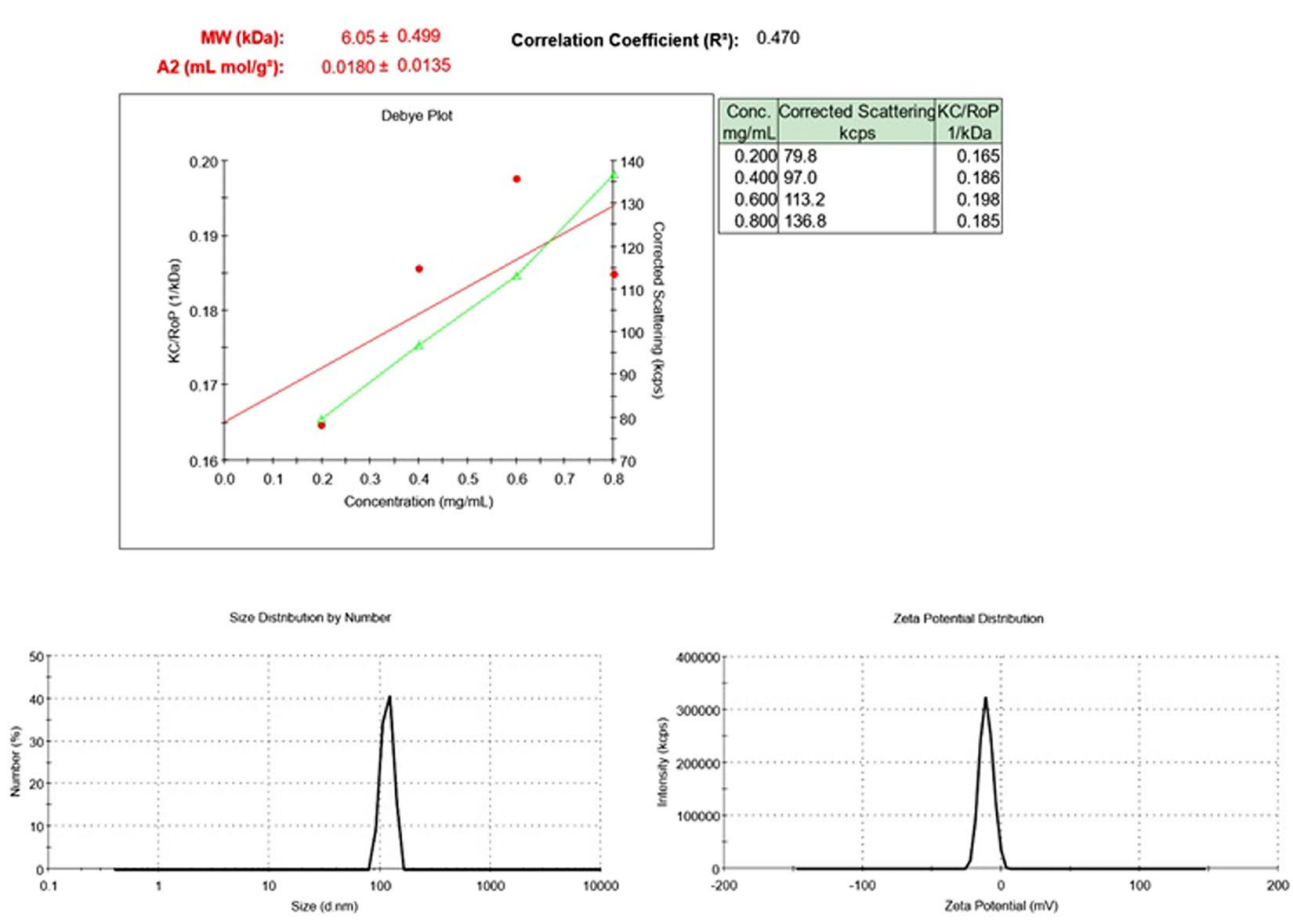
Average molecular weight, size distribution and zeta potential of the Apt-ALGDG2 conjugate after 6 months at 25 °C.

After 6 month, none of the samples which were incubated in phosphate buffered saline (PBS) at −20 °C and +4 °C have shown noteworthy changes in the color (clear), pH (6.2), average molecular weight and Zeta potential compared to the fresh sample. However, the sample which was tested at room temperature after 6 months showed substantial changes. The average molecular weight of the sample was declined (6.05 ± 0.499 kDa) and the hydrodynamic size distribution (117 nm) and zeta potential (−10.9 mV) were altered dramatically (Fig. 6).

### *In vitro* cytotoxicity assay

The results of XTT assay which evaluated the cell proliferation are depicted in (Figure S2). None of the samples (Iohexol, ALGDG2-Iohexol and Apt-ALGDG2-Iohexol) in all concentrations on both cell-lines are significantly cytotoxic 24 h after treatment. After 48 h, all concentrations of Apt-ALGDG2-Iohexol on MCF-7 cell-line, 100 µM of ALGDG2-Iohexol on MCF-7 cell line, 100 µM of Iohexol on both cell-lines and 20 µM of Iohexol on HEK-293 have cytotoxicity. Moreover, there are significant toxicities of Iohexol (100 µM and 20 µM), ALGDG2-Iohexol (100 µM) and all concentrations of Apt-ALGDG2-Iohexol on MCF-7 cells after 72 h. Interestingly, there is no cytotoxicity of Apt-ALGDG2-Iohexol after 72 h on the normal cells (HEK-293). In contrast, two concentrations of the ALGDG2-Iohexol (100 µM and 20 µM) and all of the Iohexol concentrations are toxic on HEK-293, 72 h after treatment. Remarkably, data indicate the toxic effect of conjugate on MCF-7 cells and non-poisonous impact of it upon normal cells which can lead us to use the composition as a theranostic nanomedicine.

### Apoptosis-necrosis detection

**Figure 7:**
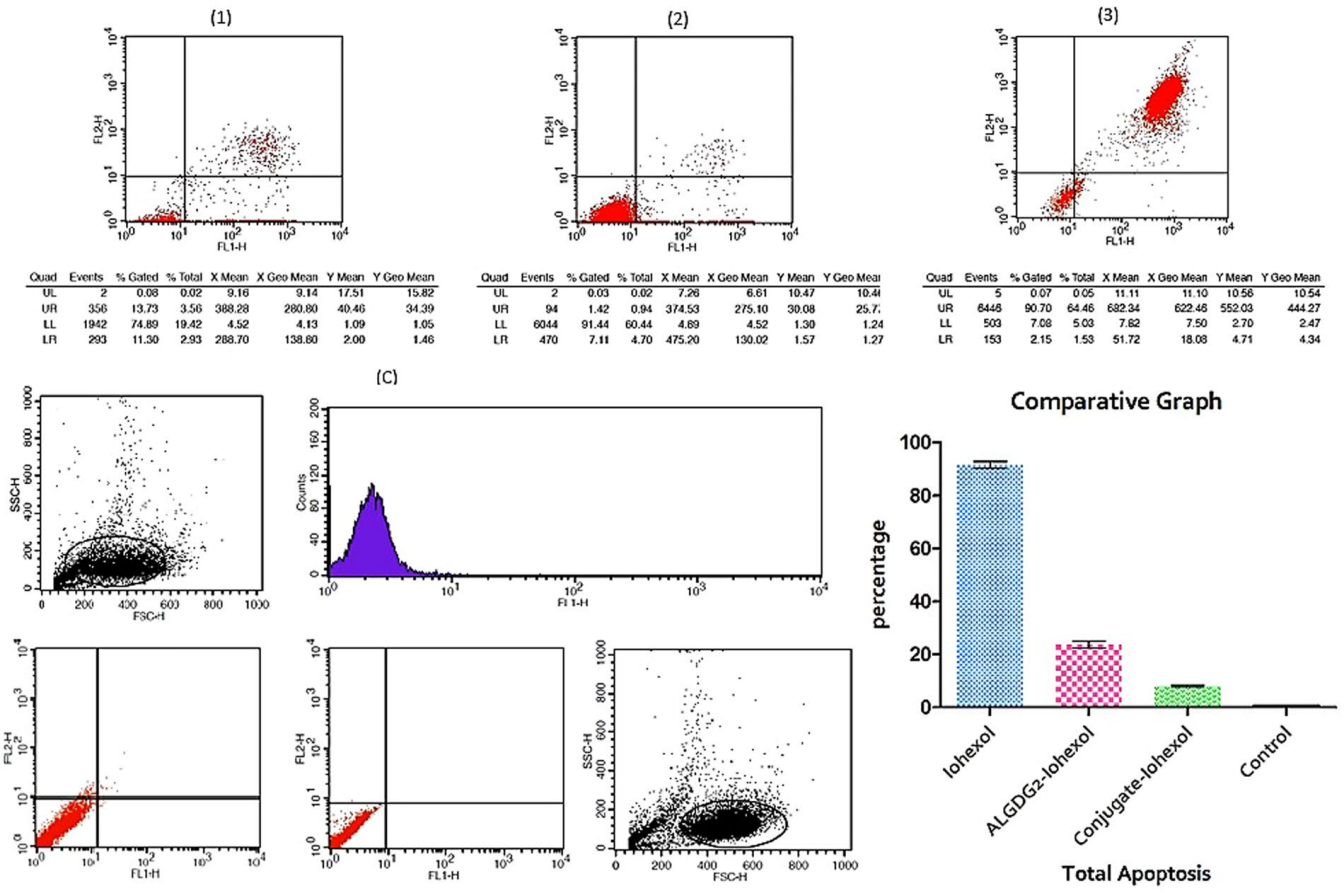
Flow cytometry and comparative graph: apoptosis/necrosis detection of HEK-293 cells (nucleolin^−^) using flow cytometry. (1) ALGDG2-Iohexol, (2) Apt-ALGDG2-Iohexol (3) Free Iohexol. (C) Controls: (right) treated unstained and (left) untreated unstained. Comparative graph shows the effect of three different compounds on the HEK-293 cells. Data are presented as mean ± SEM. Based on statistical analyses, all three components have significant differences in total apoptotic cells from the untreated control cells (p value < 0.05).

The cell population was analyzed using the signals which were detected from annexin V-FITC (FL-1) and PI (FL-2). The double negative population in the LL (lower left) quadrant (annexin V-FITC negative and PI fluorescence negative) depict the viable cell population. The early apoptotic cells are shown in the LR (lower right) quadrant, and the UR (upper right) quadrant show the late-apoptotic/necrotic cell population. The graphs are represented in (Fig. 7). As it shows in the graphs, 89.67 ± 1.03% of the HEK-293 cell population which were treated with free Iohexol are undergone late-apoptosis/necrosis, 1.9 ± 0.25% have experienced early apoptosis and only 8.35 ± 1.27% have remained healthy. For ALGDG2-Iohexol, the results are: 13.31 ± 0.42% late-apoptotic/necrotic, 10.4 ± 0.9% early apoptotic and 76.07 ± 1.18% of them are alive. Remarkably, 91.88 ± 0.44% of the cell population which were treated with Apt-ALGDG2-Iohexol have stayed healthy and fresh, only 6.64 ± 0.47% are in the early apoptotic stage and 1.455 ± 0.035% of the cells show late-apoptosis/necrosis. Data obviously confirm the result of *in vitro* cytotoxicity assay (*above*) and represent the non-toxic effect of conjugate (Apt-ALGDG2-Iohexol) in comparison with the conventional contrast media (Iohexol) and even non-targeted ALGDG2-Iohexol on normal cells.

### *In vitro* cellular uptake (quantitative and qualitative)

In this study, two different methods used to assess and compare the uptake of conjugate and dendrimer on both normal and cancerous cell-lines. (Table 1) presents the results of quantitative method via ICP-MS (Limit of quantification (LOQ) = 10 ppb). Additionally, qualitative evaluation by means of cell imaging multi-mode microplate reader confirms the previous results and shows a remarkable tendency of the desired conjugate to the cancerous cells compared to the normal cells. As it illustrates, after 4 h of treatment, plain ALGDG2 could enter MCF-7 cells as well as HEK-293 cell-line. The absorption of ALGDG2 conjugated to aptamer into the MCF-7 cells was more efficient as it displays in (Figure S3).

**Table 1:** *In vitro* cellular uptake using ICP-MS.

	IODINE (PPB)		
	Iohexol	ALGDG2-Iohexol	Apt-ALGDG2-Iohexol
MCF-7	33	145	211
HEK-293	26	80	28

### Morphological study of cells after treatment

The morphological status of the MCF-7 and HEK-293 cell lines after 72 h of treatment with Apt-ALGDG2-Iohexol, ALGDG2-Iohexol and free Iohexol compared with the controls are shown in (Figure S4). Pictures evidently show the disruptive effect of free Iohexol upon the HEK-293 cells (d) and toxic impact of Apt-ALGDG2-Iohexol upon the MCF-7 cells (b) after 72 h of treatment. The morphology of HEK-293 cells treated with ALGDG2-Iohexol (c) has changed as it is shown in the picture and some giant cells or syncytia were observed under the microscope. However, the growth of the cells did not change considerably.

### Spiral Computerized Tomography (CT) Imaging

**Figure 8:**
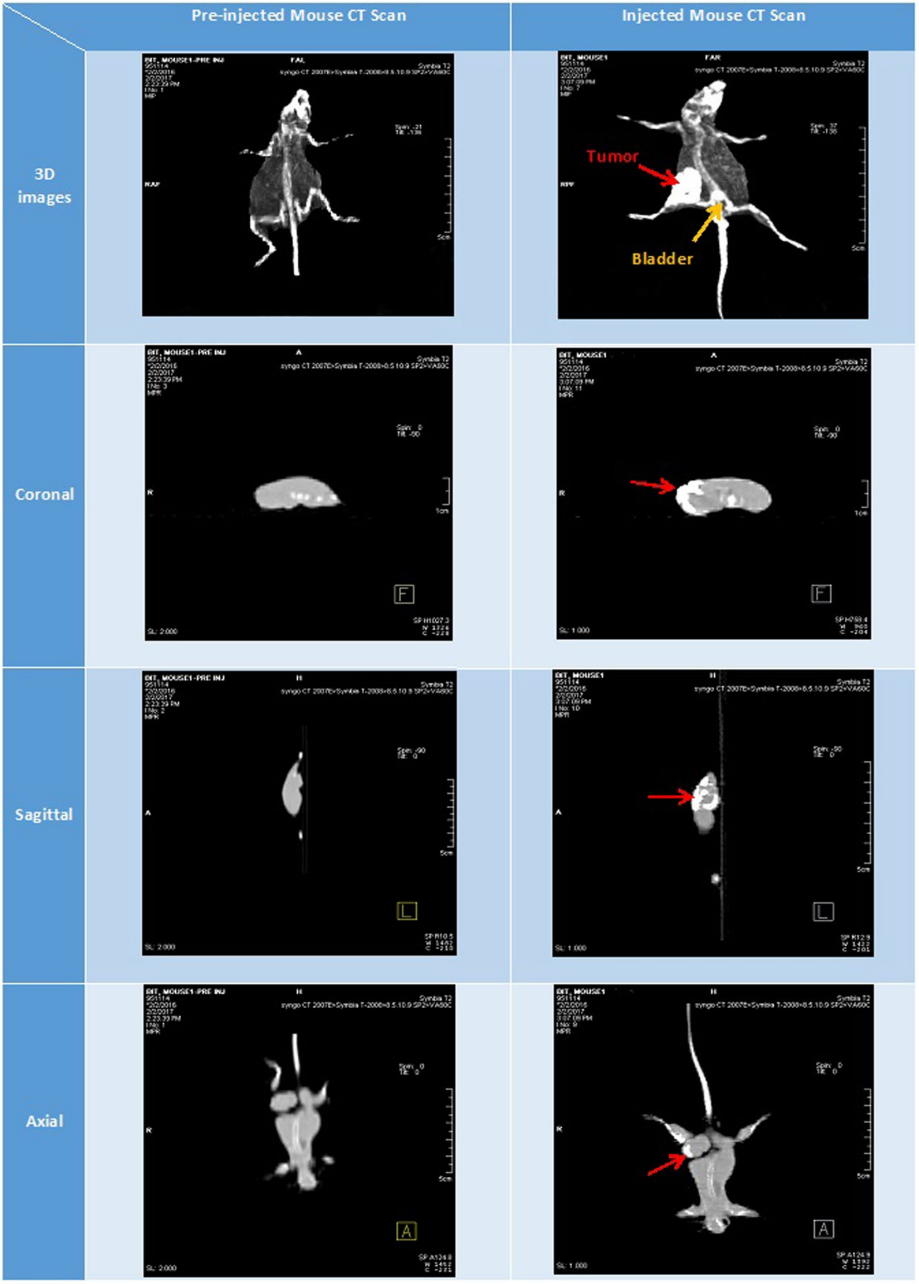
Cross-sectional spiral CT images of pre-injected and injected mouse with Apt-ALGDG2-Iohexol after 20 min. Red arrows point the tumor site in each picture. Yellow arrow shows the bladder site.

Cross-sectional CT images of mouse before and after injection of Apt-ALGDG2-Iohexol are depicted in (Fig. 8). As it clearly shows, tumor site and bladder are evident after injection, while they were invisible before injection. CT numbers of each animal’s tumor site are presented in (Table 2).

**Table 2:** CT numbers of tumor sites.

Animal No.	Minimum	Maximum	Distance (cm)
1	−274	+861	1.07
2	−94	+864	1.43
3	−36	+840	1.8

### *In vivo* safety assessments of Apt-ALGDG2-Iohexol

#### Pathology of Apt-ALGDG2-Iohexol injected 4t1 breast tumor mouse model

The microscopic findings from kidney tissue sections shows that no necrotic tubules are observed in the renal tubules in control and injected with Apt-ALGDG2-Iohexol (test) groups (Figure S5). The structure of liver tissue sections of mice in control and test groups includes the normal hepatocytes, hexagon lobules, nuclei, hepatic cords, sinusoids, portal veins, and central veins (Figure S5). The structure of spleen tissue in control and test groups is natural with no vascular and inflammatory changes. Based on histopathological findings, increased cell death in the tumor tissue is observed in Apt-ALGDG2-Iohexol treated group compared with control group and the cells’ density in the tumor tissue of treated group (b) was reduced compared with control group (a).

#### Blood test of Apt-ALGDG2-Iohexol injected normal rats

Common blood test (functionality test) used as a detection of any probable liver and kidney function abnormalities which originate from Apt-ALGDG2-Iohexol treatment shows no dysfunction in these two organs in treated rats compared to the control group and reference intervals (Table 3).

**Table 3:** Kidney (creatinine and urea) and liver (SGPT, SGOT and ALP) functionality test (common blood test).

	SGPT (ALT) (U/L)	SGOT (AST) (U/L)	ALP (U/L)	Creatinine (mg/dL)	Urea (mg/dL)
Apt-ALGDG2-Iohexol treated group	25.37 ± 0.8762	60.73 ± 6.224	110.7 ± 6.566	0.6233 ± 0.05175	41.00 ± 2.082
Control (untreated) group	20.00 ± 1.528	58.33 ± 4.096	105.7 ± 5.783	0.5667 ± 0.03756	38.67 ± 1.856
Rat Reference Intervals	17.5–30.2	45.7–80.8	56.8–128	0.2–0.8	32–45

Data analyzed by T-test followed by Mann-Whitney post-test and presented as Mean ± SEM (n = 3). All differences were statistically insignificant.

## Discussion

The exquisite goal of this study was to construct a novel molecular imaging agent with high affinity to cancer cells and low toxicity on normal cells compared to the conventional contrast medias. Another point which was considered was to develop an economical construction which is cost-effective to use frequently in monitoring the course of cancer treatment. For these purposes, anionic linear globular dendrimer G2 was prepared with additional purification steps compared to the previous methods. Then, it was confirmed and characterized via AFM, HNMR, FT-IR, LC-MS, ELS and DLS and activated by EDC to be conjugated to 5′-NH_2_-AS1411 aptamer using -COOH active groups of ALGDG2 and NH_2_ active groups of aptamer to form covalent amide bonds. Conjugation was carried out by using bath-sonicator. After purification, the conjugate was characterized using AFM, ELS, DLS and SLS techniques. The comparison between the data of ALGDG2 and the conjugate confirmed the successful conjugation and delineated the proximate efficiency of conjugation. Subsequently, Iohexol loaded to the conjugate and dendrimer respectively by the means of shaker incubator. The biological studies (cytotoxicity, uptake and morphological status determination) were performed with XTT assay and ICP-MS separately. To be more accurate in defining and confirming the results of the two abovementioned parts of biological studies, apoptosis/necrosis study and dual staining of the cells were employed respectively. The morphological changes of the cells were observed using inverted microscope. *In vivo* studies were done to assess the safety of the Apt-ALGDG2-Iohexol on mice’s bodies using pathology and blood tests. Targeting nature of Apt-ALGDG2-Iohexol was tested by spiral CT Imaging and pathology test.

Accumulation of contrast agent into the tumor site via a targeting agent can reduce toxicity, enhance efficiency and the resolution of radiographic images. Off-target cytotoxicity reduction was carried out by using a negative charged, low molecular weight, monodispersed nanoparticle (ALGDG2) which has a PEGylated core with citric acid side chains. The PEG-based nature of ALGDG2 could have increased its passive affinity to the cancerous cells and make the structure biocompatible. Moreover, its citric acid side chains made the structure biodegradable due to the fact that citric acid is one of the intermediates in the citric acid cycle (known as TCA or Krebs cycle) in the cells. Thus, ALGDG2 can degrade easily soon after entering and the derivatives can produce energy after a series of chemical reactions in the living cells^21^. Thanks to the further purification steps after synthesis of ALGDG2, DMSO was removed perfectly and pH of the composition raised up (from 4.6 to 5.8) to be less acidic and poisonous for the cells. Furthermore, utilizing a synthetic single strand DNA-based oligonucleotide (AS1411 aptamer), the construction became targeted to the cancer cells and non-immunogenic and faster cell-penetrated compared to antibodies, peptides, etc. which are kinds of biomolecules^37^. In 2004, Farokhzad *et al*.^38^ have published first report of a new bio-conjugate which consisted of poly (lactic acid)-block-polyethylene glycol (PEG) co-polymer with a terminal carboxylic acid functional group (PLA-PEG-COOH nanoparticle) and A10 PSMA aptamer (RNA aptamer) which specifically binds to prostate-specific membrane antigen, an overexpressed transmembrane on prostate cancer epithelial cells. AS1411 is a cancer specific aptamer which is in the second phase of clinical trials on behalf of patients diagnosed with metastatic renal cell carcinoma or acute myeloid leukemia and in the phase I of clinical trial for non-small cell lung cancer^39–41^. Comparing AS1411 (DNA based, 26 bp, MW: 8272.41 g/mol^42^) with other aptamers which are similar in terms of performance (targeting cancer cells), such as MUC1 (DNA based, 72 bp, MW: 22353.64 g/mole and affinity (Kd): 47.3 nM^43^) and PSMA A10 (RNA based, 56 bp, MW: 17966.62 g/mole and Affinity (Kd): 20.5 nM^44^) aptamers which were engaged in the studies before^38,45,46^, the smaller size, DNA base, Guanine-rich, quadraplex structure and expanded range of target cells for AS1411 aptamer made it more than suitable to be used as a targeting agent.

Synthesis of the two main components in the present research (aptamer and dendrimer) is straightforward and inexpensive. Hence, the price of the product will be decreasing dramatically.

Conjugation is a process which leads to form a covalent bond between the two components which are involved in the reaction. A covalent bond is an irreversible linkage which can increase the stability of the complex and decrease the inappropriate separation of the two components before entering the target cell in the serum.

This study utilized sonication method by means of a bath-type ultra-sonic cleaner. Using bath-sonicator not only could aid in coupling the aptamer and ALGDG2 in a much shorter period of time but also helped to perfectly disperse the particles even if they are agglomerated to some extent. A number of the researches exploited other methods to conjugate aptamers to nanoparticles, such as stirring or shaking the mixture gently^47–49^ but bath-sonicator reduced the reaction time from almost 2 days to only 10 minutes with high efficiency.

Besides DLS and ELS, SLS technique is used for the first time in this study for determination of the conjugate’s molecular weight and estimation of efficiency of conjugation (the average number of aptamers which are successfully conjugated to a single ALGDG2). Various other methods are usually used in researches for determining the molecular weight of a specific composition or to compare and distinct different components by assessing their molar mass, such as gel permeation chromatography (GPC)^50^ or polyacrylamide gel electrophoresis (PAGE)^51,52^. However, SLS is an accurate, simple and time-saving method for evaluating the average molar mass of nanostructures. SLS is only employing an appropriate equipment and a standard liquid (as a case in point, toluene) to use as a baseline and sample is prepared in the double deionized water which makes the procedure to be easier, faster and affordable. Additionally, data compared to the molar mass of aptamer and ALGDG2 corroborate the notion that in an average, one or two aptamers are attached to one dendrimer at a time.

One of the main challenges in the current study was the semi-adherent nature of HEK-293 cells. The key to the solution was to use XTT assay instead of MTT assay. The superiority of XTT assay over MTT assay is that XTT assay is highly sensitive, more accurate and it has a simple one-step protocol which does not require solubilization step. Thus, the semi-adherent characteristic of HEK-293 cells does not cause any problems. Cell proliferation analysis using XTT assay showed Iohexol loaded conjugates cytotoxic effects upon MCF-7 cell line 48 h after treatment. On the contrary, no significant toxic impact of the same complex has been observed on the normal HEK-293 cell-line. Moreover, data yielded by the apoptosis/necrosis assay (which determines the cause of cell death or differentiates necrotic cells from apoptotic cells via cell membrane alterations detection (such as the appearance of phosphatidylserines on the outer leaflet of the apoptotic membrane)) presents the mostly non-toxic nature of Iohexol loaded conjugate on HEK-293 after 72 h. It seems that the considerable toxicity of Iohexol loaded conjugate on cancerous cells after 48 h can be because of the anticancer effect of the AS1411 aptamer. It is reported that, AS1411 aptamer can cause cancer cell death by interfering with DNA replication via S-phase arrest^53^ and/or by making Bcl-2 mRNA stable which is a famous apoptosis inhibitor^54^.

Based on the results yielded by the two methods, Iohexol has significant toxicity on normal cells in 100 µM after 48 h of treatment and in all concentrations after 72 h. Data of apoptosis/necrosis assay confirmed the results of XTT assay on HEK-293 cells and indicated a dramatic decrease in Iohexol toxicity after loading into non-targeted ALGDG2 (ALGDG2-Iohexol) and targeted ALGDG2 (Apt-ALGDG2-Iohexol). The difference between the toxicity of ALGDG2-Iohexol and Apt-ALGDG2-Iohexol was more notable in XTT assay on HEK-293 cells after 72 h compared with the apoptosis/necrosis assay. The reason could be that similar to MTT assay, XTT assay is a colorimetric anti-proliferative assay which measures the redox potential in metabolically active cells, it can only give information about “growth inhibitory effects” and detect the number of metabolically active cells regardless of the reason. It means that, the cells which are not metabolically active, could be killed (stopped growing permanently) or stopped growing temporarily. If they were stopped growing temporarily, two fates are expected for them: (1) returning to proliferation, (2) entering the permanent cell cycle arrest stage, cellular senescence and eventually death. On the other hand, the apoptosis/necrosis assay can assess the cells’ tendency of cell death (apoptosis or necrosis).

The third frequently cause of acute kidney injury in patients is contrast induced nephropathy^55,56^. Although X-ray CT contrast medias are commonly used for imaging nowadays but have some disadvantages, such as short-time blood maintenance and non-specific bio-distribution *in vivo* which cause renal cytotoxicity^57^. In 2006, it was reported that Iohexol can cause direct dose dependent toxicity on proximal tubule cells and diminish the proliferation and viability of them concurrently. In that research, it was concluded from the results that a nonionic radiocontrast such as Iohexol cause acute renal failure^58^. In another study in 2016, acute kidney injury of Iohexol was considered and using a new complex consisted of sulfobutyl-ether-β-cyclodextrin (SBECD) and Iohexol, the toxicity decreased from 50% to 12% in a rodent model compared to the Iohexol alone^59^. Hence, it is of great importance to find an alternative to the conventional radiocontrast agents.

*In vitro* uptake of the Iohexol loaded dendrimer and Iohexol loaded conjugate compared to the free Iohexol were assessed by ICP-mass spectrometry which is a quantitative technique for detecting metallic and nonmetallic elements (certain isotopes) in a composition with high resolution. In this study, the iodine element of Iohexol is considered as the basis of uptake evaluation. Many studies made use of qualitative methods, such as fluorescent dye delivery of non-targeted nanoparticle and targeted nanoparticle to indicate the differences between the deliveries of them *in vitro* (using the term “delivery” instead of “uptake” is intentional). The present study used qualitative assessment as well. Though, the question is, are the two methods indicative of one phenomenon? The answer is no, not all the times. ICP-MS directly shows the amount of drug/agent which could be absorbed into the cells because the procedure provides the possibility of evaluating the amount of desired element from the inner part of the cell specifically. Yet, labeling the other parts of the composition (such as fluorescent modifying aptamer or nanoparticle) or delivering the dye by the distinct constructions can indicate the location of conjugate or non-targeted nanoparticle (not the drug they were carrying) and this location can be on the surface of the cell or in the cell, nevertheless it cannot distinguish the amount of drug which could not enter the cells from those which could. Thus, using a more precise and sensitive method for assessing the uptake of contrast agent beside the qualitative method using FITC and DABI was considered. Outcomes of the two methods showed remarkable differences between delivery by non-targeted and targeted dendrimer and absorption of Iohexol into the MCF-7 cells and the results presented a notable reduction in uptake of Iohexol to the normal cells via targeted ALGDG2. Conversely, it was no considerable discrepancy between the amount of Iohexol delivered using non-targeted ALGDG2 and its uptake by MCF-7 and HEK-293 cells.

*In vivo* imaging of mice injected Apt-ALGDG2-Iohexol are performed using cross-sectional spiral CT scan. Surprisingly, there was no evidence of kidneys in the images but bladder and the tumor site were obviously visible. The reason is, only the organs which have accumulated amount of Apt-ALGDG2-Iohexol in them can be visible under the CT scan and if the kidneys are not visible, it means that there is no kidneys’ absorption for Apt-ALGDG2-Iohexol and it passed through the kidneys to the bladder very swiftly. Using gold nanoparticles, different studies have been conducted to find an alternative for conventional CT contrast medias and overcome the biological barriers of them such as rapid clearance. As a case in point, P. Huang *et al*. reported a novel theranostics agent which was consisted of silica modified gold nanorods conjugated to acid folic for X-ray/CT imaging and radiation/photo-thermal therapy of gastric cancer^60^. Conversely, In the present study, an inexpensive, easier synthesized, less toxic dendrimer was utilized instead of gold nanoparticle to overcome the swift clearance and uptake issue of Iohexol and achieve more precise and high-resolution CT images.

Pathology and Blood tests were done to assess the probable toxicity of Apt-ALGDG2-Iohexol on internal organs especially kidney and liver of the animal. However, there was no considerable toxicity in those tissues. Evaluation of tumor site by pathology test showed decreased cell density and increased cell death in the animals’ tumor treated with Apt-ALGDG2-Iohexol compared to the controls. In the treated tumor site, some cells were shrinked with darker nuclei. Although, the apoptosis or necrosis stage of them are not clear.

All in all, the conjugate shows promising application in the field of molecular imaging of cancer cells. Since, it has been observed that there is a considerable toxicity of the bio-conjugate on the cancerous cell-line and *in vivo*, it can lead us to use the composition for theranostics’ purposes. Furthermore, although the conjugate appeared more appropriate to use for imaging purposes, the non-targeted dendrimer could diminish the toxicity of Iohexol on normal cells by passive delivery of it to the cancer cells. More studies can be done to evaluate the possibility of using the new compound (Apt-ALGDG2-Iohexol) for multimodal imaging. Moreover, the safe nature of the composition can encourage researchers to exploit the Apt-ALGDG2 loaded cancer therapeutics to use in potential cancer therapies.

## Methods

### Study design

All the experiments were done based on the part of the registered research proposals in Tehran University of Medical Sciences and Isfahan University of Medical Sciences. Experiments have been approved by the ethics committee of Tehran University of Medical Sciences and Isfahan University of Medical Sciences. Animals were purchased from Pasteur Institute of Iran. All the *in vivo* experiments were undertaken in accordance with World Medical Association Declaration of Helsinki (64th WMA General Assembly, Fortaleza, Brazil, October 2013).

### Anionic linear-globular dendrimer G2 (ALGDG2) synthesis

Anionic linear-globular dendrimer G2 was synthesized according to the method previously reported by Namazi, H., *et al*.^19^ and improved by Haririan, I., *et al*.^20^. Briefly, 1 mL (3.7 mmol) polyethylene glycol (PEG) 600 (Merck, Darmstadt, Germany) diluted in 10 mL Dimethyl sulfoxide (DMSO) (Merck, Darmstadt, Germany). Then, 0.75 g (2*3.7 mmol) N, N′-Dicyclohexylcarbodiimide (DCC) (Merck, Darmstadt, Germany) was added to the solution. The reaction was continued for 30 mins at room temperature while stirring. 0.71 g (2*3.7 mmol) citric acid (Merck, Darmstadt, Germany) was then added. The reaction remained stirred at room temperature for 1 h. Afterwards, 2.25 g (6*3.7 mmol) DCC and 5 mL DMSO were added and the reaction was continued under the above-mentioned conditions for about 15 mins. Subsequently, 2.1 g (6*3.7 mmol) citric acid was added and the reaction was continued for 1 week at room temperature, while stirring. After that, The ALGDG2 was filtered twice. Purification was carried out by using a Sephadex G-50 fine column (GE Healthcare Life Sciences, UK). The fraction collected was purified again by subsequent dialysis (dialysis bag 500–1000 Da cut-off) against double deionized water (D.D.W) (1 × 1 L for 2 days). The purified ALGDG2 was freeze dried (LyoTrap plus, LTE Scientific Ltd, Oldham, UK) and stored at −20 °C for future studies.

### ALGDG2 synthesis confirmation and characterizations

Synthesis of ALGDG2 was confirmed and characterized by measuring its hydrodynamic size distribution and zeta potential via dynamic light scattering (DLS) and electrophoretic light scattering (ELS) (Nano-ZS, Malvern, UK), ^1^HNMR (Bruker 500 MHz instrument), FT-IR (Perkin Elmer Spectrum BX-II spectrometer) and LC-MS (Agilent 6410 Triple Quadrupole LC/MS, Agilent Technologies, Santa Clara, CA) tests.

### Morphological study of ALGDG2

In order to compare with the conjugate’s morphological status, AFM images of the ALGDG2 were recorded in D.D.W on a microscope slide using intermittent contact (air) mode with an AFM Nano wizard II (JPK Instruments, Berlin, Germany) under ambient conditions.

### AS1411 aptamer-ALGDG2 (Apt-AlGDG2) conjugation

Aptamer-conjugated ALGDG2 was prepared as follows: ALGDG2 nanoparticles (NPs) were suspended in 500 µL of double deionized water and mixed with NHS (24 mg) and EDC (30 mg) (Sigma-Aldrich Inc., St. Louis, MO, USA). The solution was stirred for 1 h at room temperature. Unreacted EDC and NHS were removed by using subsequent dialysis for 1 h. Simultaneously, the AS1411 Aptamer (DNA based, 5′-NH2 modification supplied from TAG A/S, Copenhagen, Denmark; sequence: 5′- NH2-(GGTGGTGGTGGTTGTGGTGGTGGTGG)-3′) was dissolved in 500 µL of deionized water and heated up to 85 °C for 2 mins. After reaching room temperature, it was added to the reacted NPs (molar ratio: 1:1) and the reaction continued for 10 mins sonicated in a bath-sonicator. Finally, the unreacted aptamers were removed by dialysis against deionized water at room temperature for 2 h. The product was freeze dried and stored in −20 °C for further studies.

### Apt-ALGDG2 characterization and conjugation confirmation

#### Characterization using dynamic and electrophoretic light scattering

Apt-ALGDG2 conjugate was characterized by calculating its hydrodynamic size distribution and zeta potential via dynamic light scattering (DLS) and electrophoretic light scattering (ELS) (Nano-ZS, Malvern, UK). The sample was measured at 25 °C at a wavelength of 633 nm with He-Ne laser. Simultaneously, the size and zeta potential of dendrimers were measured under the abovementioned conditions. The comparison between these two, confirmed the successful conjugation of aptamer to dendrimer.

#### Morphological study using Atomic force microscope

With the aim of comparing the morphological changes after conjugation, the AFM images of Apt-ALGDG2 were recorded using intermittent contact (air) mode with an AFM Nano wizard II (JPK Instruments, Berlin, Germany) at 25 °C.

#### Molar mass measurement using static light scattering

With the purpose of measuring the molar mass of Apt-ALGDG2 conjugate, static light scattering (SLS) technique was performed using Zetasizer ZSP (Nano-ZSP, Malvern, UK) instrument as follows: five different concentrations of the conjugate with unknown molar mass (1, 0.8, 0.6, 0.4 and 0.2 mg/ml) were prepared in deionized pre-filtered water at 25 °C. Then the samples were filtered with 0.22 micron filter and the scattered light intensity of each sample was measured and the correlation coefficient (R^2^), molecular weight (kDa) and 2^nd^ virial coefficient (A2) (mL mol/g^2^) were reported respectively. The comparison between the conjugate’s molecular weight and identified NH_2_-modified AS1411 aptamer’s molecular weight (measured using MALDI-TOF Mass Spectrometry by TAG A/S company) and dendrimer’s molecular weight (reported previously^61^) confirmed the conjugation and indicated the approximate efficiency of the conjugation process.

### Iohexol loading

Iohexol (Santa Cruz Biotechnology Inc., Texas, USA) loading into the Apt-ALGDG2 conjugates and ALGDG2 were obtained by mixing Iohexol with each sample (5 mg excessive Iohexol and 10 mg ALGDG2-conjugated aptamer (standard molar ratios: ~1 mg Iohexol: 10 mg ALGDG2-conjugated aptamer and 1 mg Iohexol: 2 mg dendrimer)) and incubating them while shaking at 4 °C for 10 mins using an orbital shaker/ incubator (unimax 1010/ incubator 1000, Heidolph Instruments, Germany). Unentrapped excess Iohexol was removed by dialysis (dialysis bag 500–1000 Da cut-off) in deionized water (1 × 1 L for 2 days).

Entrapment/encapsulation efficiency (EE%) which is considered as the drug percentage that is successfully entrapped/ absorbed into the nanoparticle was calculated by microplate reader (BioTek Instruments, USA) at 254 nm as follows:

Different concentrations of Iohexol were dissolved in double deionized water to illustrate the standard curve. Then, the data collected from the unentrapped Iohexol which is solved in the water while dialysis used to measure entrapment efficiency by applying the equation below:

$$ \% {\rm{EE}}=[\frac{\mathrm{drug}\,\,{\rm{added}}-{\rm{unentrapped}}\,{\rm{drug}}}{{\rm{drug}}\,{\rm{added}}}]\,\ast \,100$$

### Conjugate Stability analysis

In order to assess the stability of the conjugate, pH, molar mass and zeta potential of separate samples dissolved in phosphate-buffered saline (PBS) or D.D.W were studied under different conditions (at −20 °C, room temperature (25 °C) and 4 °C) for 6 months.

### *In vitro* cytotoxicity assay

The nucleolin^+^ human breast cancer cell line (MCF-7) and nucleolin^−^ human embryonic kidney cell line (HEK-293) were acquired from national Cell Bank of Iran (Pasteur Institute, Tehran, Iran). The cells were cultured with DMEM and PRMI-1640 mediums, each supplemented with 10% fetal bovine serum (FBS) in 5% carbon dioxide (CO_2_) humidified incubator. The cytotoxicity of Apt-ALGDG2-Iohexol, ALGDG2-Iohexol and free Iohexol were assessed at 100, 20, 4, 0.8, 0.16 µM concentrations using tetrazolium-based colorimetric (XTT) test using cell proliferation kit II (XTT) (Roche Applied Science, Germany), 24, 48 and 72 h after treatment via microplate reader (BioTek Instruments, USA) at 475 nm.

### Apoptosis/necrosis detection by flow cytometry

In order to assess the extent of apoptosis or necrosis of HEK-293 cells, Annexin-V-FLUOS staining kit (Roche Diagnostics, Penzberg, Germany) was exploited using the protocol described by Lakshmanan, I., *et al*.^62^. Briefly, a total 4 × 10^6^ HEK-293 cells were seeded in the 6-well cell culture plate as described above. After 24 h, 3 × 10^6^ of cells were treated with 100 µM of Apt-ALGDG2-Iohexol, ALGDG2-Iohexol and free Iohexol and the rest remained untreated as control and all were incubated for 72 h. After incubation, the media of the cells were collected into the separate 15 ml polystyrene tubes. Then, the cells were trypsinized, collected and centrifuged, washed with PBS and then re-suspended to stain with Annexin-V and pI by the kit to perform apoptosis/necrosis detection via flow cytometry. 10^4^ cells per sample were obtained, then analyzed using Cell Quest software.

### *In vitro* cellular uptake

#### Quantitative assessment using Inductive Coupled Plasma- Mass Spectrometry

Uptake assessments of Iohexol loaded ALGDG2, Iohexol loaded conjugates and free Iohexol were performed via Inductive Coupled Plasma - Mass Spectrometry (ICP-MS) (Elan 6100 DRC-e, Perkin-Elmer, USA) by measuring the amount of absorbed iodine by cells using the method described by Kim, Chaekyu, *et al*.^63^. Briefly, Apt-ALGDG2-Iohexol, ALGDG2-Iohexol and free Iohexol were incubated with pre-seeded MCF-7 and HEK-293 cell lines (25,000 cells/well) in 24 well plates for 5 h. Afterward, the cells were washed 3 times with PBS and then 300 µl lysis buffer was added to the cells and the cell lysate remained digesting by means of HNO_3_ and H_2_O_2_ in 3:1 concentration ratio overnight. Subsequently, 3 ml aqua regia was added to the samples and reaction continued for 3 more hours to be completed. After that, the samples were diluted to 100 ml deionized water and the sample solutions were measured by ICP-MS.

#### Qualitative assessment using cell imaging multimode microplate reader

The qualitative cellular uptake of non-targeted and targeted ALGDG2 by MCF-7 and HEK-293 cells were studied using cell imaging multi-mode microplate reader (Cytation 3, BioTek, USA). Cells were cultured in a 24-well plate under 5% CO_2_ and 95% relative humidity at 37 °C. After 24 h, the medium was changed and the cells were treated with 100 µM FITC (as fluorescence dye) loaded targeted and non-targeted ALGDG2 for uptake measurement and incubated for 4 h at 25 °C. Subsequently, the cells were washed 3 times with PBS and fixed with paraformaldehyde (4% for 15 min). After incubation, the cells were washed 3 times again with PBS. The cells were stained with DAPI (0.5 mg/ml) for 3 mins to staining the nuclei and then washed 3 times with PBS. The images of each sample were then recorded and compared to one another.

### Morphological study of cells after treatment

Morphology of the two cell-lines treated with Apt-ALGDG2-Iohexol, ALGDG2-Iohexol and free Iohexol was checked out via inverted microscope (LABOMED TC400, USA).

### Spiral Computerized Tomography (CT) Imaging

Three 6–7 weeks old, 20–25gr female BALB/c mice were exploited for primary 4t1 tumor establishment based on the protocol described in *current protocols in Immunology* ^64^. The tumor diameter measured by Vernier caliper every 3–4 days. After tumors’ appropriate growth to about 10 to 18 mm, the anesthetized 24–28 gr mice were injected with equivalent volume doses (0.1 ml per 25gr of each mouse body weight) with Apt-ALGDG2-Iohexol (1.6 µM) intravenously in the tail vein and horizontally placed under the SPECT/CT system (Symbia T2; Siemens Medical Solutions USA, Inc.). Cross-sectional images were acquired before injection and 20 mins after injection. Then, images were analyzed using SYNGO software.

### *In vivo* safety assessments of Apt-ALGDG2-Iohexol

#### Pathology of Apt-ALGDG2-Iohexol injected 4t1 breast tumor mouse model

Two 4t1 tumor mouse models (prepared as mentioned previously) were injected intravenously in their tail vein with Apt-ALGDG2-Iohexol (concentration: 1.6 µM as described above) every 18 hours for 48 hours. Two 4t1 tumor mouse models were used as controls without injection. Subsequently, each mouse was sacrificed and the chest cavity was opened with dissecting equipment to expose the internal organs. Afterward, the animal’s kidney, spleen, liver and tumor tissues were harvested for histopathological examinations. Tissues were fixed in formalin 10% (Merck, Germany) for 72 h and then dehydrated through a graded-alcohol series (70, 80, 90, 95 and 100%). Then, tissues were cleaned in two changes of xylene and impregnated with two changes of molten paraffin wax. The samples were embedded and blocked out in paraffin and sectioned at 5 μm slides. After sectioning, the slides were stained with hematoxylin and eosin (H&E). slides were monitored under light microscope and the photomicrographs of them were obtained for evaluation of tissue degeneration to assess the toxic effects of the novel nano-theranostics on the animal’s body and targeted nature of it to the tumor site.

#### Blood test of Apt-ALGDG2-Iohexol injected normal rats

Normal white Wistar Rats (n = 3, 10–12 weeks old, 250–300 gr) were both used as test (T) and control (C) as follows. The T group were injected with Apt-ALGDG2-Iohexol (1.6 µM, 1.5 ml per 400 gr) intravenously. The C group were the same animals injected with placebo (animals before injection with Apt-ALGDG2-Iohexol). Before injection and after 12 hours of injection, blood samples were collected and tested for SG OT/PT, ALP, creatinine and urea amounts in the rats’ bodies to evaluate the kidney and liver function (Rat reference intervals are based on *Exotic companion medicine handbook for veterinarians* ^65^).

### Statistical analysis

Descriptive and inferential data analysis were done using Prism 5 and excel software (Microsoft Office 2013). Significant differences were analyzed by the means of T-test and one-way ANOVA followed by the Tukey test to cluster comparison. Graphs were drawn by Prism 5. All quantitative data are presented as mean ± SEM. P < 0.05 was considered statistically significant.

## Electronic supplementary material

Supplementary Information
