# Supplementary material for: AS1411 Aptamer-Anionic Linear Globular Dendrimer G2-Iohexol Selective Nano-Theranostics
Source: Sci Rep. 2017 Sep 19;7:11832. doi: 10.1038/s41598-017-12150-8 (PMC5605695; doi:10.1038/s41598-017-12150-8)
Supplement: Supplementary file 1 — Supplementary Information [file 41598_2017_12150_MOESM1_ESM.pdf]

AS1411 Aptamer-Anionic Linear Globular Dendrimer G2-Iohexol Selective Nano-Theranostics

Pardis Mohammadzadeh[1] , Reza Ahangari Cohan[2,*,+], Seyedeh Masoumeh Ghoreishi[3], Ahmad Bitarafan-Rajabi[4] and Mehdi Shafiee Ardestani[5,*,+]

[1] Department of Genetics and Molecular Biology, Isfahan University of Medical Sciences, Isfahan, Iran.

[2] Department of Pilot Nanobiotechnology, New Technologies Research Group, Pasteur Institute of Iran, Tehran, Iran.

[3] Department of Radiopharmacy, Faculty of Pharmacy, Tehran University of Medical Sciences, Tehran, Iran.

[4] Echocardiography Research Center, Cardiovascular Interventional Research Center, Department Of Nuclear Medicine,

Rajaie Cardiovascular Medical And Research Center, Iran University Of Medical Sciences, Tehran, Iran.

[5] Department of Radiopharmacy, Faculty of Pharmacy, Tehran University of Medical Sciences, Tehran, Iran.

[*]**Corresponding**. Dr. Mehdi Shafiee Ardestani, Pharm.D, Ph.D

shafieeardestani@tums.ac.ir

[+]These authors contributed equally to this work

AS1411 Aptamer-Anionic Linear Globular Dendrimer G2-Iohexol Selective Nano-Theranostics

Pardis Mohammadzadeh[1] , Reza Ahangari Cohan[2,*,+], Seyedeh Masoumeh Ghoreishi[3], Ahmad Bitarafan-Rajabi[4] and Mehdi Shafiee Ardestani[5,*,+]

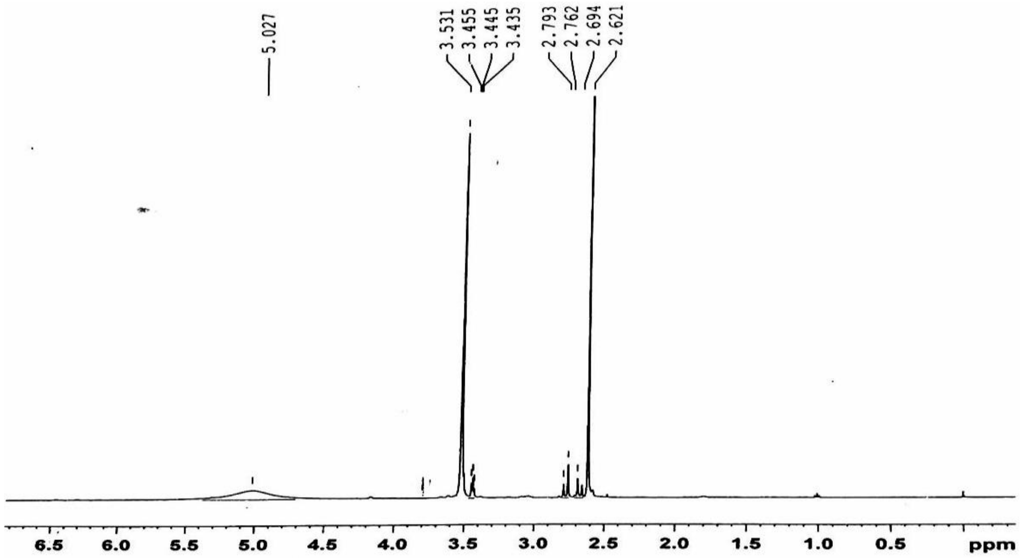

S1.a. ^{1}HNMR spectrum characterization of anionic linear globular dendrimer G2. Strong peak at the area of 3.8 shows the PEG structure (O–CH2–CO). The A–B hydrogen system in citric acid was observed at the area of 2.8 and 3 ppm with doublet peaks. Presence of PEG structure in anionic linear globular dendrimer G2 was approved by the triplet peaks of O-CH2-CH2 (3.4 ppm).

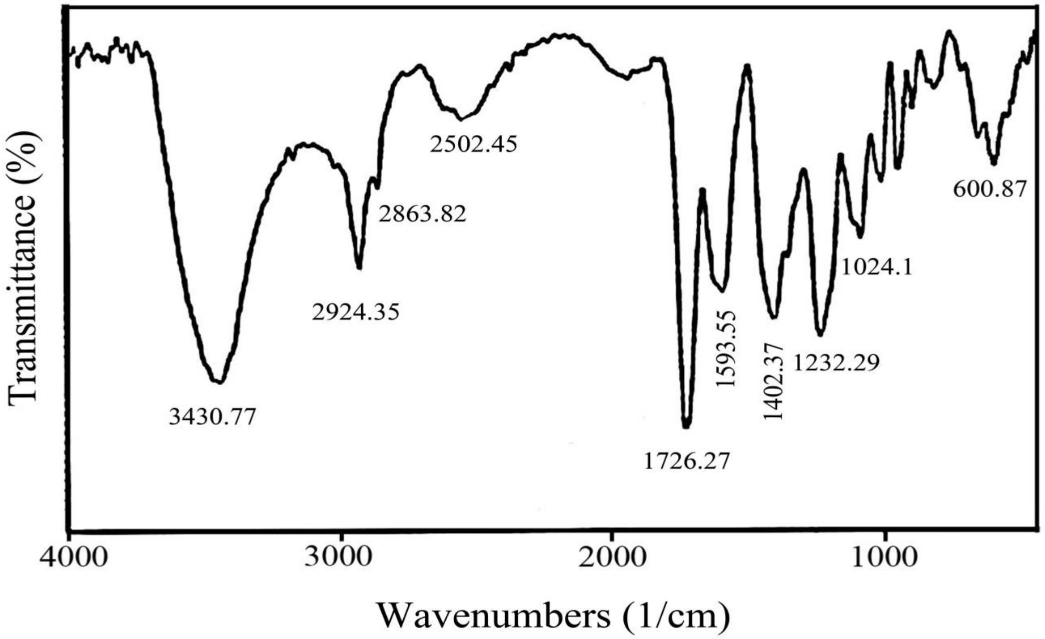

S1.b. FT-IR spectrum of the anionic linear globular dendrimer G2. (C-O) in steric bonds which are the representative of anionic linear globular dendrimer G2 synthesis by the means of citric acids between first generation and second generation of the dendrimer is shown at 1232 cm^{-1}. (O–H) stretches which are observed in 3430 cm^{-1} are assigned as citric acids. Furthermore, (C=O) bonds of terminal citric acids of G2 dendrimer observed in 1726 cm^{-1}.

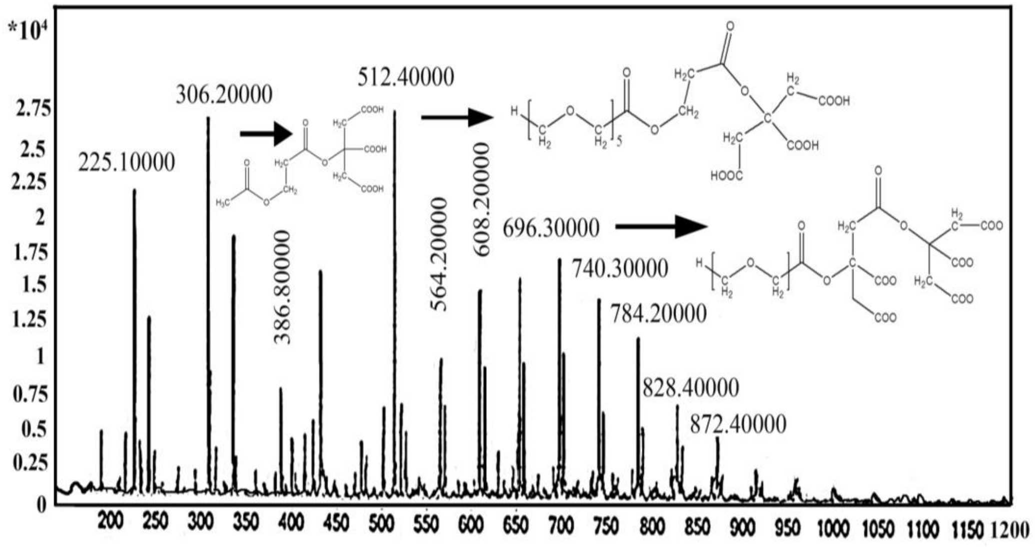

S1.c. LC/MS graph for the accurate synthesis confirmation of anionic linear globular dendrimer G2. The obtained chemical structure is in agreement with our approximation.

Figure S1. Characterizations of anionic linear globular dendrimer G2 by ^{1}HNMR (S1.a), FT-IR (S1.b) and LC-MS (S1.c). Explanations are presented blow each figure (S1.a, S1.b and S1.c).

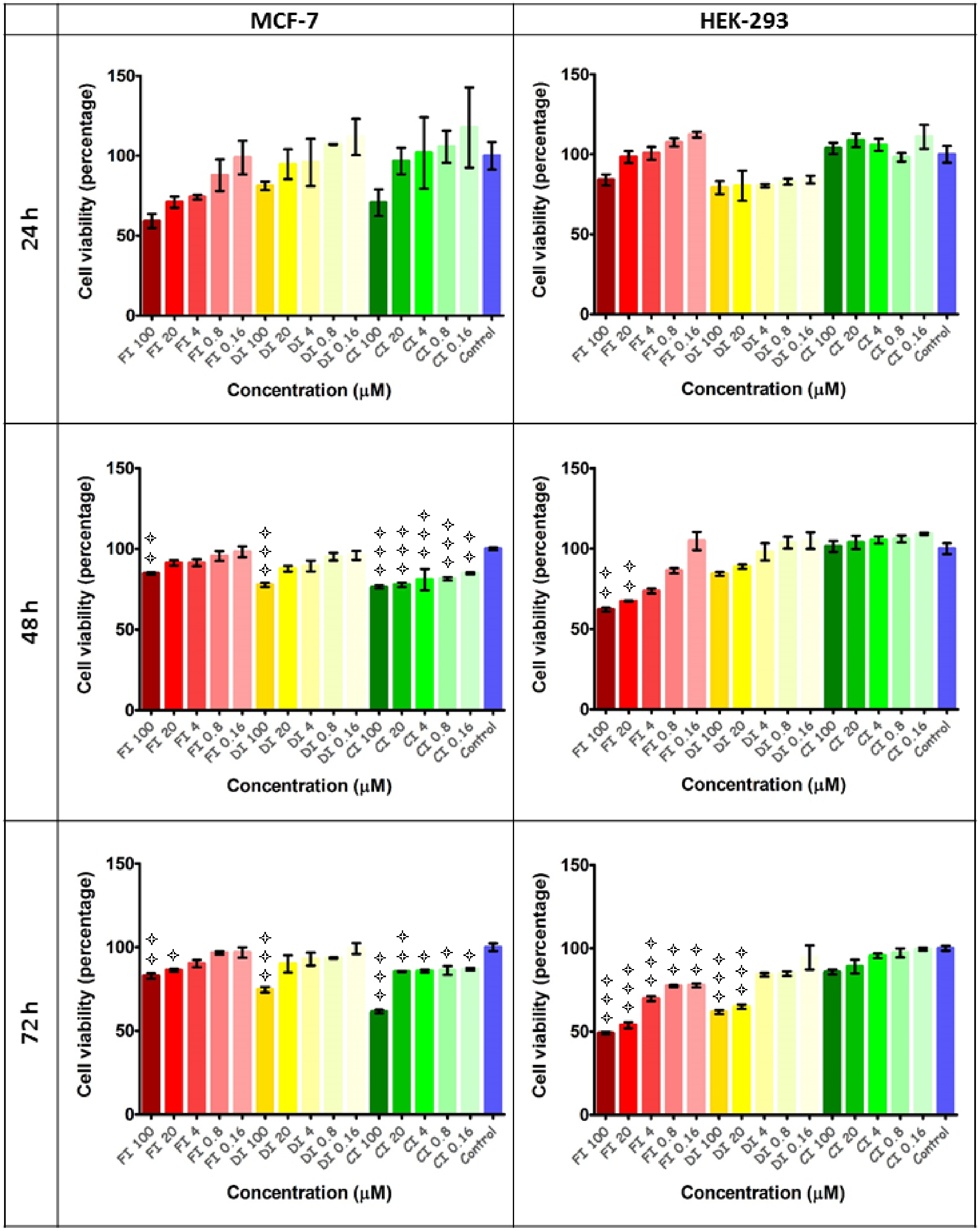

Figure S2. Effect of free Iohexol (FI), ALGDG2-Iohexol (DI) and Apt-ALGDG2-Iohexol (CI) on the viability of MCF-7 and HEK-293 cell-lines. Data are presented as mean ± SEM (n = 3) in each bar. Data analyzed by one-way ANOVA with Tukey post-test. Significant difference from the untreated control cells at $p < 0.05$, $p < 0.001$ and $p < 0.0001$ are shown by the asterisks *, **, *** respectively.

AS1411 Aptamer-Anionic Linear Globular Dendrimer G2-Iohexol Selective Nano-Theranostics

Pardis Mohammadzadeh[1] , Reza Ahangari Cohan[2,*,+], Seyedeh Masoumeh Ghoreishi[3], Ahmad Bitarafan-Rajabi[4] and Mehdi Shafiee Ardestani[5,*,+]

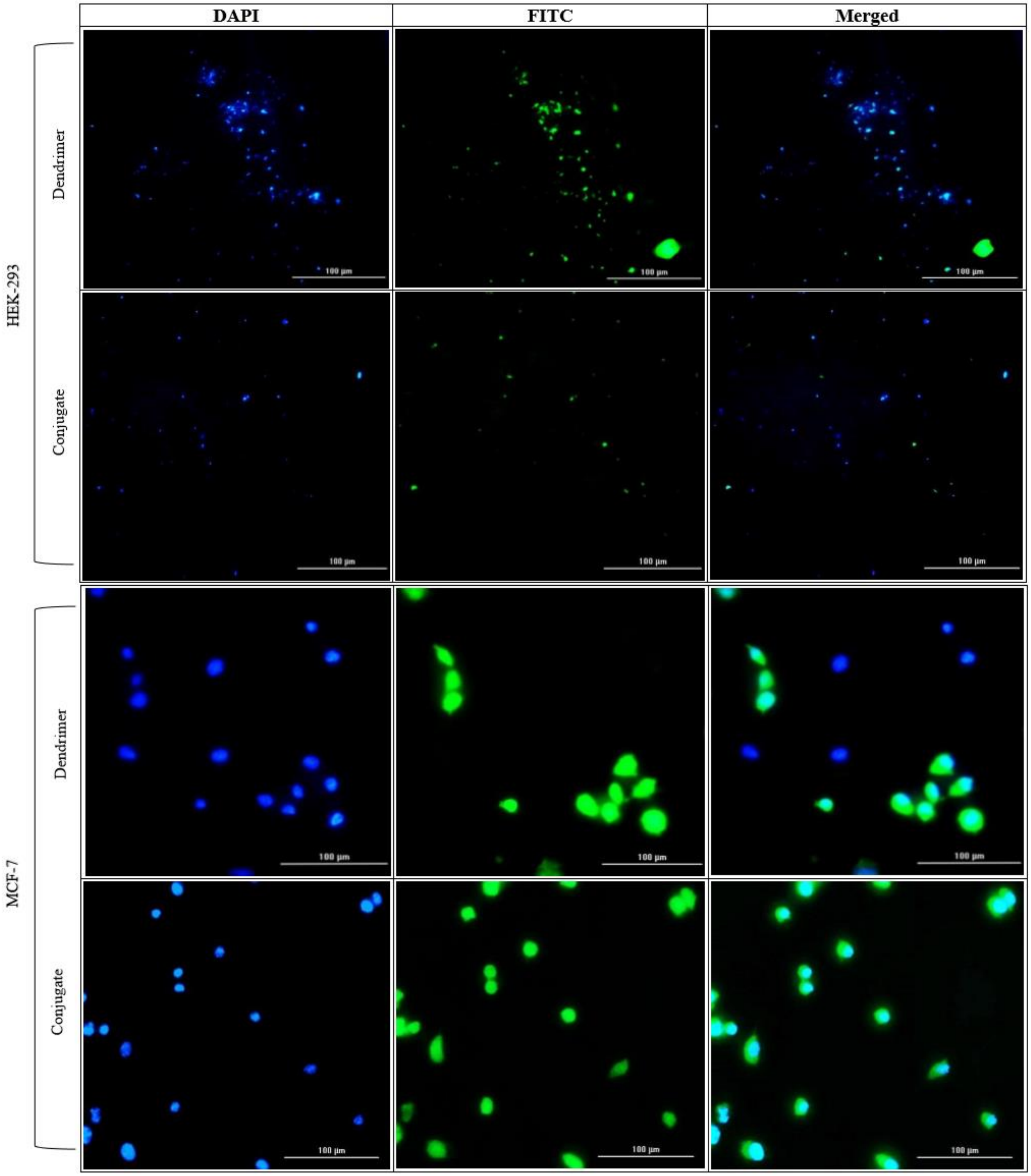

Figure S3. Qualitative assessment of *in vitro* cellular uptake via cell imaging multi-mode microplate reader (magnification of 20x).

A

B

C

D

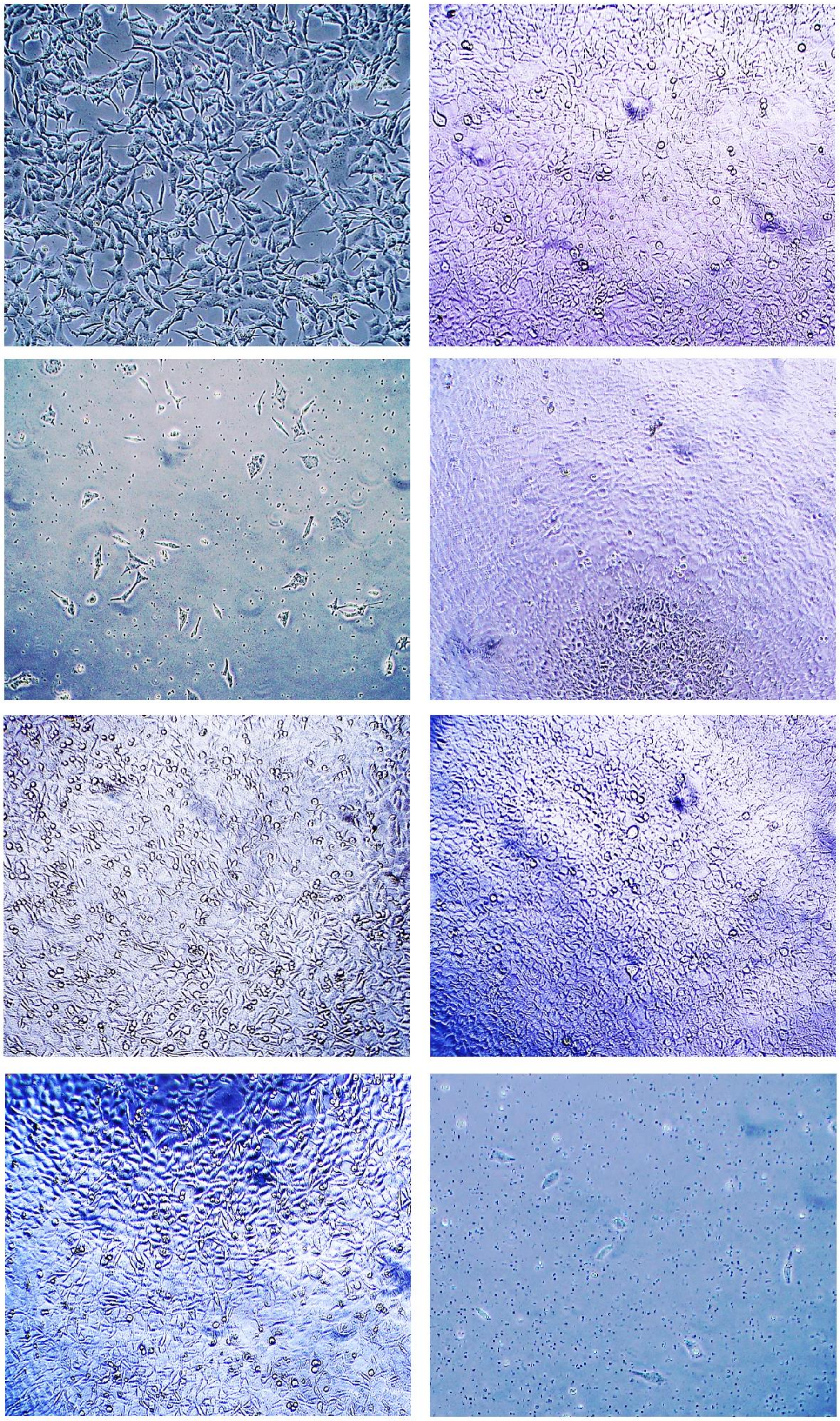

Figure S4. Inverted microscope images of MCF-7 (left) and HEK-293 (right) cells: a) Untreated (control) b) Iohexol loaded conjugates c) Iohexol loaded ALGDG2 and d) Free Iohexol.

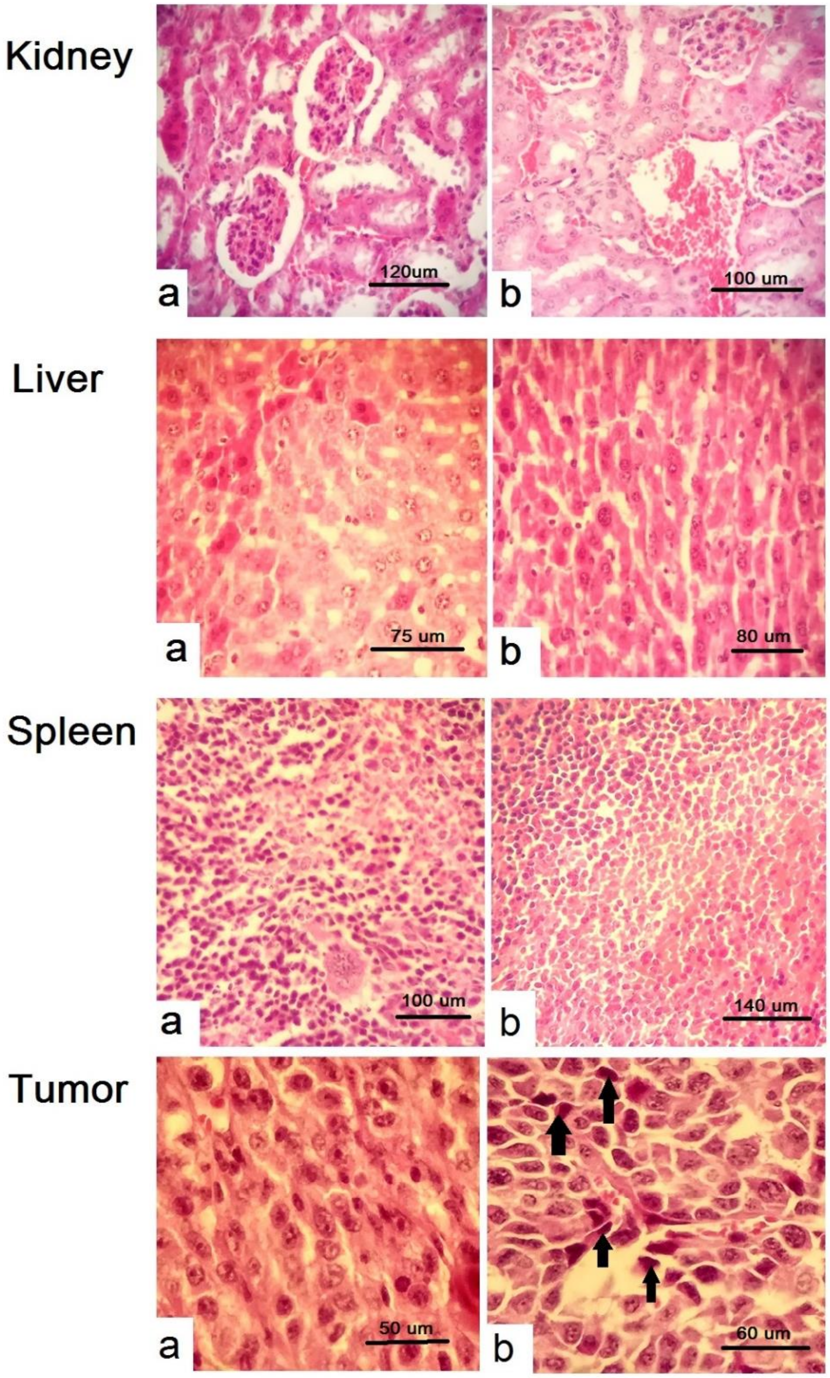

Figure S5. Histopathological evaluation of Apt-ALGDG2-Iohexol *in vivo*. Based on microscopic findings of spleen, liver, kidney, and tumor tissues from control (a) and test (Apt-ALGDG2-Iohexol treated) (b) mice no necrotic tubules are observed in the renal tubules in control (a) and test (b) groups. No congestion and other pathological changes were observed in the liver tissue. In the spleen, no vascular and inflammatory changes were observed in control (a) and test (b) groups. In the tumor tissue, increased cell death were observed in test group (b) compared with control group (a). (Arrows show the dead cells) (Magnification for all the images: 400X).
